# Insight into Post-Pandemic Needs in Healthcare and Well-Being Among Francophone Families in the Canadian Prairies

**DOI:** 10.3390/ijerph23020167

**Published:** 2026-01-28

**Authors:** Catelyn Keough, Marianne Turgeon, Elyse Proulx-Cullen, Anne Leis, Danielle de Moissac, Kristan Marchak, Sedami Gnidehou

**Affiliations:** 1 Campus Saint-Jean, University of Alberta, Edmonton, AB T6C 3N2, Canada; ckeough@ualberta.ca (C.K.); mturgeon@ualberta.ca (M.T.); kmarchak@ualberta.ca (K.M.); 2 Department of Health Sciences, Faculty of Medicine, University of Saskatchewan, Saskatoon, SK S7N 5E5, Canada; proulxcullen.elyse@usask.ca; 3 Department of Community Health and Epidemiology, Faculty of Medicine, University of Saskatchewan, Saskatoon, SK S7N 5E5, Canada; anne.leis@usask.ca; 4 Faculty of Science, University of Saint-Boniface, Winnipeg, MB R2H 0H7, Canada; ddemoissac@ustboniface.ca; 5 Department of Medical Microbiology and Immunology, Faculty of Medicine, University of Alberta, Edmonton, AB T6G 2E1, Canada; 6 School of Public Health, University of Alberta, Edmonton, AB T6G 1C9, Canada

**Keywords:** language minority setting, Canadian Prairies, Francophone families, COVID-19, health and well-being, post-pandemic needs

## Abstract

**Highlights:**

**Public health relevance: How does this work relate to a public health issue?**

- The COVID-19 pandemic has exacerbated the pre-existing disparities in information and service access in French for these populations.
- This work demonstrates the lack of equal access to healthcare and social services between the official languages of Canada in a language minority context.

**Public health significance: Why is this work of significance to public health?**

- This work provides insight into the lived experiences of Francophone families throughout the pandemic and the needs that remain to be addressed.
- Working directly with Francophone families provides results that can improve community belonging, healthcare, and education of this population.

**Public health implications: What are the key implications or messages for practitioners, policy makers, and/or researchers in public health?**

- Our results highlight common and province-specific priorities to improve policy and service planning in a bilingual country.
- This study provides access to solid research-based evidence to inform tailored responses, such as enhancing mental health services in AB and SK and fostering Francophone community and educational support in MB.

**Abstract:**

Francophone populations outside Quebec were disproportionately affected by the COVID-19 crisis. Despite French being one of Canada’s official languages, access to information and services in French remains limited. This study examined Francophone families’ (FF) post-pandemic health and well-being needs (PPHW) in the Canadian Prairie provinces. An online survey assessed PPHW needs among 319 FF in Alberta (AB), Saskatchewan (SK), and Manitoba (MB). Respondents ranked PPHW needs from a predefined list; logistic regression analyzed socio-demographic influences. Divided into AB/SK and MB cohorts, sociodemographic profiles were statistically distinct for many variables, but with similarities found in gender of respondents (women: 73% in AB/SK, 79% in MB), marital status (married: 81% in AB/SK, 88% in MB), area of residence (urban: 86% in AB/SK, 81% in MB), and number of children (2 children: 49% in AB/SK, 41% in MB). Three high-priority needs were shared across provinces: (1) access to recreational, athletic, and artistic activities in French for children (variations by child gender); (2) access to French healthcare professionals (variations by education level and language difference); and (3) social activities in French for families. AB/SK respondents prioritized mental health services in French for adults and youth. MB families prioritized belonging to a Francophone community (variations by gender of children) and education services in French (variations by age of children). Understanding these common and province-specific priorities can inform policy and service planning.

## 1. Introduction

The Severe Acute Respiratory Syndrome Coronavirus 2 (SARS-CoV2) pandemic, commonly known as COVID-19, has had a devastating impact worldwide. It created an environment of uncertainty, stress, and even despair [1,2]. In Canada, the COVID-19 pandemic has resulted in over 4.6 million cases and 60,871 deaths since its onset in March 2020 [3,4]. Similarly to most countries, in addition to the direct health impacts this pandemic has posed in Canada, new threats to families emerged as a result of social isolation, including feelings of fear and anxiety, school and childcare closures, financial and employment insecurity, housing instability, and changes to health and social care access [5,6,7,8]. For the latter, studies have reported that technological barriers, limited availability of providers and community resources, perceived lack of services, and gaps in communication were commonly noted challenges to accessing virtual Canadian primary care and mental health services [9,10,11].

Interestingly, studies have demonstrated that, in Canada, several minority groups dealt with significant health and well-being inequities during the COVID-19 pandemic. For example, immigrant and refugee populations faced amplified mental health challenges, inequities because of social determinants of health, and decreased access to primary care and community resources due in part to a lack of awareness and cultural differences between providers and patients [12,13,14]. Limited access to information also led to a higher rate of SARS-CoV-2 exposure and hospitalization among immigrants [15,16]. While many of them experienced fear and anxiety for several reasons during the pandemic [14,17], those who were newcomers especially faced multiple disruptions in their healthcare due to limited access to material resources and a lack of social support [18,19,20]. Feelings of marginalization were also perceived within these vulnerable groups [20,21,22]. Similar concerns about mental health, lack of social support, and feelings of marginalization were also reported in the 2SLGBTQ+ community [21,23]. This raised some concerns about inequities in health and well-being in several different vulnerable minority populations, among which official language minority communities should be included.

The Canadian healthcare system is accessible to all residents and protected by the Charter of Rights and Freedoms [24]. Specifically, the Canada Health Act ensures that all Canadians are entitled to some health services (such as primary care, immunizations, and hospital services). Other services are provided by extended healthcare services, depending on provincial policy (such as mental health and fertility treatments). However, the Canada Health Act does not provide explicit information about the language of service. It is well-established that language plays a key role in access to healthcare and well-being services, which include access to information, preventative services, diagnosis, and treatment [25,26]. In Canada, a bilingual and multicultural country with English and French as official languages [27,28], members of Official Language Minority Communities (OLMCs) often face language barriers and limited services in their minority language, which undermines their access to health and social services, as well as family support programmes [29,30]. OLMCs are defined as “groups of people whose preferred official language is not the language of the majority in their province or territory” [31], which include Francophones living outside of Quebec and Anglophones living in Quebec. OLMCs, particularly Francophones living in the Prairies, represent about 8.4% of the population in Manitoba and 4.7% in Saskatchewan (2021), making them smaller and more geographically dispersed than Francophone communities in Ontario (11.1%) or New Brunswick (41.9%). Because of their small size and dispersion, Prairie OLMCs face unique challenges: language barriers, limited supply of services in the minority language, and gaps in access to culturally appropriate health, social, and family support services, particularly in rural areas—a phenomenon that is well documented [32,33]. Despite progress, several lines of evidence have highlighted the inability to access social services in the official minority language of one’s community as a risk factor for poor well-being [34,35]. For Francophones outside Quebec, studies highlight barriers related to misunderstandings about health conditions [33,36], challenges in finding French-speaking health professionals [37,38,39], emotional distress and discontent with care provided, as well as hesitation to seek care due to language barriers [37,40].

While Anglophones living in Quebec also experience significant linguistic and cultural barriers to accessing social services, English is significantly predominant in Canada [41]. Furthermore, the probability of finding an English-speaking health and/or social services provider in Quebec is higher than finding a French-speaking corresponding services provider outside Quebec [29,42]. As such, Francophone OLMCs living in language minority settings in Canada represent a unique population group that needs to be further explored.

Taken together, we considered that Francophones residing in predominantly English-speaking regions in Canada may have experienced language-related COVID-19 challenges. In a previous publication [43], we reported for the first time that more than 55% of respondents from FF with children under 19 years old living in the Canadian Prairies (Alberta, AB; Saskatchewan, SK; and Manitoba, MB) had been negatively impacted by the COVID-19 pandemic. Challenges reported by these participants included supporting the mental health of adults and their children, maintaining adequate schooling, accessing health and social services in French, and having to fend for themselves [43]. While our studied population was composed of a high proportion of French-speaking immigrants who have been described as being at particular risk of health and well-being challenges [44,45], we also identified that differences in official language proficiency predicted difficulties in accessing health and social services. Surprisingly, these challenges did not predict the perceived negative and positive impact of the COVID-19 pandemic on FF [46]. However, our understanding of these FF priority post-pandemic healthcare and well-being needs (PPHW) remains limited.

Key factors such as the number of Francophones, their geographical origin, the number of healthcare and well-being services provided in French, as well as perceived access to healthcare, can impact healthcare and well-being priorities [41,47,48]. This article aims to report on the PPHW needs of FF residing in a minority context in the Canadian Prairies with a specific focus on these relevant variables as well as other risk factors. To our knowledge, this study is the first to define, using a survey-based quantitative method, healthcare and well-being needs for this geographic region, as well as province-specific priorities. Risk factors influencing these needs and sociodemographic variables, which could be significant predictors of healthcare and well-being needs, were identified. Contributions of this work include painting a portrait of the Canadian Prairies and their prioritization of needs following the COVID-19 pandemic, as well as the province-level contrasts within this region.

## 2. Materials and Methods

### 2.1. Study Sites

The chosen study sites include three Canadian Prairie provinces: AB (population of 4.7 million), SK (population of 1.2 million), and MB (population of 1.4 million) [41]. These were selected for geographic and Francophone demographic reasons. According to the 2021 census, the proportion of the population able to speak French was 6.2% in AB, 4.7% in SK, and 8.4% in MB. Differences were observed in origin, with most Francophones in AB originating from elsewhere in Canada (48%) or abroad (28%) as compared to SK (29% and 18%, respectively) and MB (13% and 15%, respectively) [41]. Of note, MB provides comparatively greater community and healthcare support for Francophone OLMC than other Prairie provinces. For example, Shared Health—MB’s provincially mandated health agency—coordinates the delivery of bilingual clinical and preventive care across the province. This is achieved through the designation of bilingual facilities and programmes, the implementation of staff training and retention strategies to maintain French-language proficiency and cultural competency, and the ongoing collection of data to inform policy development aimed at supporting the Francophone population [47]. Finally, a study has reported that the proportion of Francophones who perceived difficulty in obtaining services in the minority language was similar and relatively high in AB (67%) and SK (72%) compared to a lower value in MB (40%) [48]. Given the greater similarity between the sociodemographic profile of Francophones and service provision in AB and SK, the research team decided to combine data from these provinces and compare them with MB data [46].

### 2.2. Participants, Inclusion Criteria, and Sampling

Participants, inclusion criteria, and sampling technique are described in detail elsewhere [43]. Briefly, we recruited Francophone adults (≥18 years old; male and female) who lived either in AB, SK, or MB at the time of study. In this study, Francophones were self-identified. Participants were excluded if they were not currently living in one of the three provinces, if they were not a parent, grandparent, or legal guardian of children aged 0–19 years old, or if they did not answer the two eligibility questions. FF were invited to participate in a 15 min online survey distributed by Francophone community partner organizations, members of a pan-Canadian network of the Société Santé en français, whose mandate is to make social and health services accessible in French from coast to coast (Réseau Santé Alberta, Réseau Santé en français de la Saskatchewan et Santé en français Manitoba). The survey was open between March 2023 and May 2023 and was made available via QR (Quick Response) code on posters displayed at community organizations and university billboards and shared through social media, email, word of mouth, a Francophone radio station, and FF, known to the researchers. An event at Campus Saint-Jean (University of Alberta) provided participants with Internet access and tablets to complete the survey. Upon completion, participants from each province could enter a draw to win an iPad.

### 2.3. Survey Questionnaire

Details related to the survey creation, pre-testing, validation, and implementation are described elsewhere [43,46]. A secondary-level, four-part electronic questionnaire (SurveyMonkey hosted by the University of Saskatchewan) was composed of 22 questions [43]. One survey question focused specifically on participants’ post-pandemic needs, which was extracted and analyzed for this study. This question presented a list of 13 needs, inspired by data collected at World Café discussion groups led by our research team [43], and aligned with previously established research objectives. Participants were asked to rank their four highest-priority needs from this list. Additionally, the socio-demographic section, which contained 14 questions, was considered. It included factors such as proficiency in French and English, age and gender of participants and children, immigration status, presence of a support system in one’s home province, education level, and marital status.

### 2.4. Data Sources and Processing

Data sources and processing have been described elsewhere [43]. Briefly, participants were excluded from initial recruitment due to ineligibility (*n* = 291). Thus, data from 319 respondents were considered for analysis [43].

### 2.5. Measures of Priority Post-Pandemic Healthcare and Well-Being Needs

Thirteen items related to PPHW needs were selected, which include all thirteen potential needs listed in the survey questionnaire (see Appendix A). Participants were asked to rank from 1 to 4, in order of importance (with 1 being the most important and 4 being the least important), their four priority needs from the given list, and answers were assessed by recoding the original ranking system as a binary variable of “Priority need” or “Not a priority need”; variables ranked 1 and 2 were considered a “Priority need”, whereas variables 3 and 4, and those not selected, were ranked “Not a priority need”. This recoding choice presents a trade-off during analysis, reducing the granularity of the original ranking information and instead captures whether a need was prioritized rather than its relative importance. However, analysis using a binary variable allows us to conduct logistic regression and calculate Odds Ratios for our variables. Frequency of responses of indicated needs was then calculated for both province groups (AB/SK and MB). On the basis of the World Health Organization, healthcare (HC) and well-being (WB) were defined, respectively, as “[enabling] health systems to support a person’s health needs—from health promotion to disease prevention, treatment, rehabilitation, palliative care and more” and is “a resource for daily life and is determined by social, economic and environmental conditions” [49,50]. Three items fit the definition of HC, whereas 10 items fit the definition of WB. We then identified the five priority needs for each province group separately (determined by the highest frequencies). Three priority needs that were common to both cohorts were identified.

### 2.6. Statistical Analysis

Statistical analysis was conducted using JASP Version 0.19.1.0 software [51]. For descriptive statistics, percentages and frequencies were used. Contingency tables with *p*-values were used to determine statistically significant differences (*p* < 0.05) in socio-demographic variables between participants from AB and SK and those from MB. Student’s *t*-test was performed to compare the mean scores between cohorts across each of the 13 post-pandemic need items. Prior to analyses, assumptions of normality and homogeneity of variances were assessed. Where the Brown–Forsythe test indicated a violation of the equal variance assumption (*p* < 0.05), Welch’s *t*-test was used to adjust for unequal variances. To examine associations between variables/factors, the Odds Ratio (OR, the cross-product ratio of 2 by 2 table entries) and the Wald test were performed. Confidence interval (CI) and statistical significance were set at 95% (*p* ≤ 0.05), respectively. Variables analyzed include sociodemographic factors such as participant age, immigration status, education level, urban vs. rural setting, language proficiency level, presence of support system in one’s home province, marital status, as well as number, age, and gender of children. These factors were considered given their relevance to the objectives of the study. Descriptive statistics and analyses examining associations between socio-demographic factors and post-pandemic needs using Odds Ratios were exploratory in nature. Finally, to confirm adequate detection of differences, a power analysis in G*Power 3.1 showed that the pooled AB/SK comparison with the MB subgroup (*n* = 192 and *n* = 127) was powered to detect small-to-medium effects (d = 0.25–0.30 and OR ≈ 1.4–1.6), assuming 80% power and alpha = 0.05.

## 3. Results

### 3.1. Study Population

**Figure 1:**
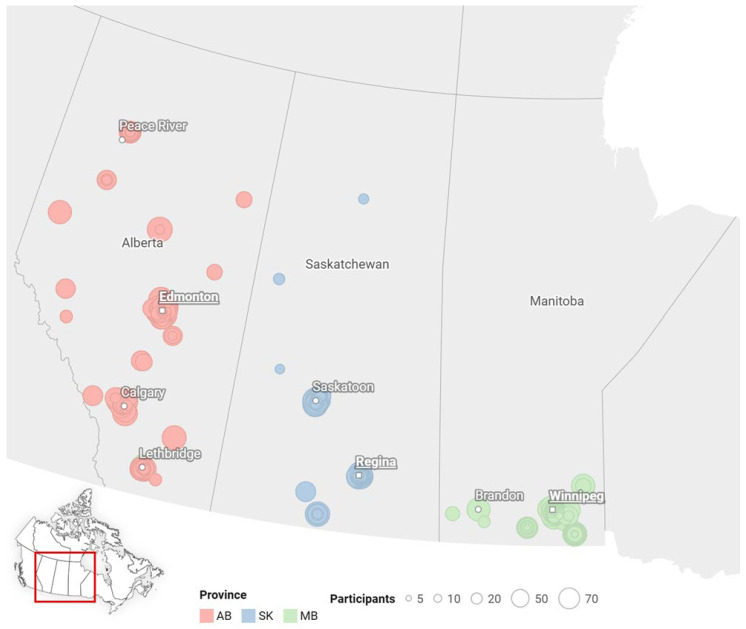
The geographic distribution of participants across the Canadian Prairies.

The general characteristics of the two cohorts are shown in Table 1. Participants across both cohorts shared several demographic similarities. The majority were Francophone women from both immigrant (AB/SK = 49%; MB = 39%) and non-immigrant (AB/SK = 49%; MB = 61%) families, most of them married (AB/SK = 81%; MB = 88%). The most prevalent family type was two children (AB/SK = 49%; MB = 41%), many of school age (AB/SK = 63%; MB = 50%). Of the 319 respondents, 84% were primarily situated in urban or centralized areas, especially in MB, whereas respondents were more widely dispersed in AB/SK (Figure 1). However, several distinctions emerged between cohorts. Contingency tables reveal significant differences in participant age (*p* = 0.03), education level (*p* = 0.01), immigration status (*p* = 0.003), presence of a support system (*p* = 0.001), language difference (*p* = 0.01), and family type, including age (*p* = 0.03) and gender (*p* < 0.001) of children.

### 3.2. Post-Pandemic Healthcare and Well-Being (PPHW) Needs

#### High-Priority Shared PPHW Needs

We considered that despite the differences in sociodemographic profiles between the two cohorts, FF with children and youth across the three Prairie provinces could share various major PPHW needs. To test this hypothesis, we first measured the post-pandemic needs frequency in our study population using 13 statements related to HC and WB (Appendix A) with definitions taken from the World Health Organisation (see Section 2). Using calculated frequencies, we observed only one need with a significant difference between cohorts, which is access to information via the media in French (*p* = 0.004) (Appendix A). Secondly, we identified common high-priority HC and WB needs by using the frequency of each post-pandemic need and selecting the top 5 for each province group. As expected, these high-priority PPHW needs were shared between cohorts. They are access to recreational, athletic and artistic activities for children and youth (28.6% of participants indicating this need in AB/SK and 23.6% in MB), access to healthcare professionals in French (21.3% in AB/SK and 29.9% in MB), and social activities in French for families (18.2% in AB/SK and 22% in MB) (Table 2).

A logistic regression analysis was performed to test if any of these three priority needs were associated with sociodemographic data. Significant associations were observed between the need for recreational, athletic, and artistic activities for children/youth, as well as the gender of children (Table 3). Families with only girls were 3.00 times (OR = 3.00; *p* = 0.006) more likely than the reference group of families with only boys to report this need. Additionally, families with both girls and boys (mixed-gender) were 2.37 times more likely than boys-only families (OR = 2.37; *p* = 0.04) to indicate this same need. Furthermore, participants with an undergraduate degree/post-secondary diploma (OR = 2.55; *p* = 0.01) or a graduate degree (OR = 2.81; *p* = 0.002) were over two times more likely to indicate a need for healthcare professionals in French compared to those with a high school education or less. We found that participants who were English-dominant were 3.17 times more likely than English/French balanced participants to want access to healthcare professionals in French (OR = 3.17; *p* = 0.048). No significant associations are found between sociodemographic variables and the shared need for the third priority, which is social activities in French for families (Table 3).

### 3.3. Province-Dependent High-Priority Healthcare and Well-Being Needs

To determine whether the differences in needs between cohorts are associated with sociodemographic variables, we assessed the specific needs in each group by considering unique needs only. In AB/SK, access to mental health services in French for adults (16.7% of participants indicating this need) and for children/youth (16.1%) were reported as specific post-pandemic needs (Table 2). These needs are considered as HC-focused using the definition from the World Health Organization, as they “enable health systems to support a person’s health needs” [49]. We observed a trend in the data whereby younger participants (20–35 years old) were more likely to indicate mental health services in French for children and youth as a need compared to older age groups, though these values did not reach significance (Table 4). No significant associations were found between tested sociodemographic risk factors and access to mental health services in French for adults (Table 4).

Remarkably, while FF reported two unique HC-related needs in AB/SK, two distinct WB post-pandemic needs were identified in MB: a need to feel part of a Francophone community (17.3%) and a need for quality education services in French (17.3%) (Table 2). These needs fit in the definition of WB as they are “determined by social, economic, and environmental conditions”, as per the World Health Organization’s definition [50]. Interestingly, families with both girls and boys appear 5.88 times less likely to indicate the need to feel part of a Francophone community (OR = 0.17; *p* = 0.03) compared to the reference group with boys only (Table 5). For the unique need for education services in French, there is a trend observed that participants aged 36–45 are approximately five times more likely to indicate this need than those aged 20–35 years, although the values are not significant. Another trend to be considered, although non-significant, is that families with ≥3 children are nearly eight times more likely to indicate a need for education services than participants with one child. A significant association was also found between this need and participants with preschool-aged children, as being 7.76 times more likely to indicate this need than the reference group of families with school-aged children (OR = 7.76; *p* = 0.04)

## 4. Discussion

This study, a first of its kind among Canadian Prairie Francophone families, examined both shared and region-specific post-pandemic healthcare and well-being (PPHW) needs, underlining the influence of sociodemographic factors on these priorities. To note that data from AB and SK were examined together in comparison to those from MB, given the greater similarity between the sociodemographic profiles and service provisions in the grouped provinces [46]. Notably, our findings revealed that families with certain child gender compositions, higher education levels, and English-language dominance were more likely to report common needs across provinces. However, the pattern diverged when looking at unique needs: in AB/SK, younger participants showed a trend of indicating specific needs in mental health, whereas in MB, families with preschool-aged children and with boys only were significantly more likely to identify distinct needs, whereas younger participant age showed only a trend. These differences point to the importance of tailoring interventions to regional, demographic, and family composition contexts. It is important to note that, given the exploratory nature of our analyses, effect estimates are interpreted cautiously, particularly where subgroup sizes are small.

### 4.1. Demographic Information

Considering our unique study population across the Canadian Prairies, it is important to acknowledge how sample composition may influence the interpretation of findings. A large proportion of participants being women and individuals from urban areas can be explained by recruitment methods, with the survey being more easily publicized through urban networks and organizations. Moreover, women are disproportionately more likely to engage in social research, particularly in surveys related to health or family well-being [52]. Also, Francophones in urban settings are more likely to have reliable internet and digital literacy, increasing the likelihood of completing online surveys compared to those in rural regions [53]. More than 80% of respondents from both populations being married is rooted in a regional cultural norm in the Canadian Prairies, which historically shown high marriage rates since 1991 [54]. Focusing on Francophone populations, cultural, linguistic, and community ties are strong; this sense of community fosters their interest in responding to research surveys relevant to them. Recognizing these factors is essential, as they provide important context for understanding the patterns observed in PPHW needs across regions.

### 4.2. Shared Needs in AB/SK and MB

FF in AB/SK and MB expressed a shared need for recreational, athletic, and artistic activities for children and youth, possibly to sustain French language skills and cultural identity among youth in a minority-language setting through structured activities [48]. Such opportunities are limited, and the COVID-19 restrictions disrupted an already limited network of French programmes, therefore heightening the urgency to restore and expand them in a post-pandemic context [55]. This need is more likely to be indicated by families of girls only or mixed-gender children (boys and girls). One possible explanation may relate to parental approaches to child-rearing, including differences in stress levels dependent on child gender, and how protective they are towards their children. A study by Negraia et al. [56] found that fathers experience greater stress when parenting all girls and mixed-gender children as opposed to only boys, whereas mothers reported greater stress when parenting all girls compared to all boys. Equally, both scenarios are a result of parents seeking shared leisure or play-focused activities for their children, especially for girls [56]. Similarly, prior research has observed a consistent relationship between parent encouragement and the amount of outdoor play of only girls, suggesting a link between parents of only girls and a need for organized activities for their children to aid in this encouragement [57]. In the previously mentioned study, it is revealed that parents are more likely to allow boys to play outdoors compared to girls, because they are more protective of their daughters compared to their sons, and therefore are possibly more inclined to seek structured and controlled recreational settings for their daughters [57,58].

FF highlighting a need for healthcare professionals in French is no surprise, considering that many Canadian provinces, including the Prairie provinces, face unmet healthcare needs due to problems such as lack of availability of services, acceptability, or accessibility barriers, including cost and transportation. Interestingly, in a study by Sibley and Glazier [59], these unmet needs were more common among women and those with higher education levels, which is partially similar to our study findings. Our analysis suggests that the need for healthcare professionals speaking French is amplified for individuals with a higher education level, primarily guided by higher odds of self-informing about health topics and seeking help for health conditions such as COVID-19 [60].

The finding that English-dominant participants are more likely to indicate the need for HC professionals in French was unexpected and may be due to the use of a convenience sample. In other words, people who signed up to complete a study about Francophone experiences during COVID-19 may feel strongly about services for this population, in general, even if they personally can make use of services in English. Future research should address this question.

### 4.3. Unique Needs in AB/SK

It is clear that the pandemic increased mental health challenges among youth, manifesting through anxiety, depression, and social isolation [61]. This was especially true in rural settings due to geographic and transportation challenges, as well as a lack of mental health providers [62]. The unmet demand for access to mental health services for children and youth, specifically in AB/SK, is therefore rational. A Canadian study on psychological responses to COVID-19 similarly reported rates of probable post-traumatic stress and generalized anxiety among both Francophone and Anglophone populations across and outside Quebec [63], reinforcing our findings on the post-pandemic need for mental health services.

Risk factor analysis revealed a non-significant trend that young parents (<36 years old) are more likely to indicate a need for mental health services for children and youth. Generally, younger parents have a lower socioeconomic status (SES), education level, and income level, meaning more parenting stress, leading them to be less able to manage their children’s emotional and behavioural concerns. They typically have a lower threshold for interpreting behaviours as problematic, deeming mental health services as a reasonable solution [64]. Younger parents may also endure what is called “dual developmental tasks”, comprising their own major life transitions such as completing education, securing stable employment and marriage, while simultaneously raising children, resulting in increased stress and reduced emotional bandwidth for children’s mental health needs, alluding to a higher perceived need for such services for their children [65,66]. It is unclear why no associations were found between sociodemographic variables and the need for mental health services for adults; further investigation is required.

### 4.4. Unique Needs in MB

Feeling part of a Francophone community is a post-pandemic priority for respondents in MB. Because Franco-Manitobans had stronger, law-mandated community resources before the pandemic, compared to somewhat weaker ones in AB/SK (see Methods), the health restrictions had a greater impact on their ability to express their Francophone identity and maintain their strong sense of cultural community [47]. The indications of this need are aimed at returning to a state of normalcy and reviving a pre-existing sense of belonging that was strongly reinforced in MB, especially. Additionally, our findings suggest that it is less likely that families with boys and girls indicate the need to feel part of a Francophone community, compared to families with only boys. According to Houndetoungan et al. [67], parents of same-gender children tend to form approximately 15% more social links compared to mixed-gender families. Relating this information to our study, it is possible that boys-only families orient parents to more cohesive, single-network engagements such as the Francophone community, as they are more likely to participate in similar activities such as sports, clubs, or school programmes. Comparing this to mixed-gender families, the possible difference in activities for girls and boys may diminish a family’s need for a single cohesive Francophone community, as they pour their identity into multiple facets.

Given that the need for quality education services in French is more evident for families with preschool-aged children, it is reasonable to hypothesize that parents may still be in the decision-making process about which programmes their child will attend and, therefore, more strongly value accessibility to these resources compared to families that are more experienced and knowledgeable about the school system.

### 4.5. Current Possibilities and Level of Services

There is a link to be drawn between the highlighted post-pandemic needs and the current state of services offered to Francophone populations in the Canadian Prairies. Province-specific findings could be useful to outline practical implications for service providers and policymakers working in minority-language contexts. In AB/SK, specifically during the period of the study (March to May 2023), there were mental health services offered in French to both adults and children through the Institut Guy-Lacombe de la Famille (AB), the Réseau santé Alberta, TAO Tel-Aide (SK), and the Réseau Santé en français de la Saskatchewan, yet community members in our study mentioned these services as post-pandemic priorities [68,69]. In the same period, Francophone communities in Manitoba were supported through the Manitoba Francophone Immigration Network and the Francophone Affairs Secretariat [70,71]. Quality education services existed through the Franco-Manitoban School Division, offering student support programmes and ensuring that education in French was available for those who sought it out [72]. Although these resources exist for FF in their respective provinces, our results highlight post-pandemic needs that are left unfilled. Therefore, it may be valuable to question the accessibility of these services for our target population, as many families may not be aware of their existence.

Importantly, our collaborative foundation with Francophone community health networks across AB, SK, and MB is a prime example of knowledge translation in action. Their involvement as stakeholders in our study population ensures that FF have access to solid research-based evidence to inform tailored responses, such as enhancing mental health services in AB and SK, and fostering the Francophone community and educational support in MB. This partnership ensures that research evidence directly guides programmes reflecting the realities and priorities of FF in the Prairies.

### 4.6. Limitations

Our study has certain limitations. First, there was a lack of male voices as 76% of respondents were women (Table 1). This underrepresentation of men may have influenced the perspectives captured, particularly of how health and well-being needs differ by gender or by family role. Another factor that may have influenced the generalizability of results is a strong representation of urban participants (84%; Table 1): although the sample size of rural participants is small, it is still sufficiently powerful to use in analysis. The convenience-based nature of sampling could also be viewed as a limitation. Additionally, we chose to use the difference score between fluency in French and English to emphasize a common effect expected in the French-speaking communities of interest. However, in using this difference, we were not able to assess the impact of a person’s fluency in each official language. Finally, comparing the ethno-linguistic characteristics of participants was not addressed, which could have added further depth to the interpretation of the findings.

## 5. Conclusions

Overall, our study underscores the importance of targeted, culturally anchored interventions to address the diverse needs of Prairie Francophone families in a post-pandemic context, specifically in terms of access to healthcare and promotion of well-being. PPHW needs vary by region and demographic profile, such as families with girls or mixed-gender children and individuals with a higher education level across the Canadian Prairies. Regionally, AB/SK respondents, especially younger parents and rural residents, prioritized children’s mental health services, while MB respondents emphasized restoring a sense of belonging to the Francophone community, especially in families with two children or only boys. Specific contributions of this work include painting a portrait of the Canadian Prairies and their prioritization of needs following the COVID-19 pandemic, as well as the province-level contrasts within this region.

## Figures and Tables

**Table 1:** The general characteristics of the study population.

Sociodemographic Variable	Alberta and Saskatchewan ^1^ (*n* = 192) Frequency (%)	Manitoba (*n* = 127) Frequency (%)	*p*-Value *
Gender			0.45
Woman	141 (73.4)	101 (79.5)	
Man	46 (24.0)	23 (18.1)	
Not specified (NA) ^2^	5 (2.6)	3 (2.4)	
Age (years)			0.03 *
20–35	26 (13.5)	33 (26.0)	
36–45	101 (52.6)	52 (40.9)	
46+	63 (32.8)	40 (31.5)	
Not specified (NA)	2 (1.0)	2 (1.6)	
Highest level of education			0.01 *
Secondary or less	53 (27.6)	20 (15.7)	
Undergraduate Degree/Diploma	70 (36.5)	43 (33.9)	
Graduate	63 (32.8)	62 (48.8)	
Other (NA)	6 (3.1)	2 (1.6)	
Marital status			0.20
Married and non-separated	156 (81.3)	112 (88.2)	
Unmarried, separated, divorced, widow or widower ^3^	31 (16.1)	14 (11.0)	
Other (NA)	5 (2.6)	1 (0.8)	
Immigration status			0.003 *
Permanent Resident or waiting for PR	48 (25.0)	22 (17.3)	
Canadian Citizen and First Nations, Inuit, or Métis	4 (2.1)	18 (14.2)	
Canadian Citizen (by birth but non-Indigenous)	91 (47.4)	60 (46.5)	
Canadian Citizen (by naturalization)	47 (24.0)	27 (21.3)	
Other	2 (1.0)	0 (0.0)	
Area of residence ^4^			0.25
Urban	165 (86.5)	103 (81.1)	
Rural	27 (14.1)	24 (18.9)	
Support system in your province			0.001 *
Yes	79 (41.1)	79 (62.2)	
No	63 (32.8)	21 (16.5)	
Sometimes	41 (21.4)	21 (16.5)	
Not specified (NA)	9 (4.7)	6 (4.7)	
Language difference ^5^			0.01 *
French-dominant	70 (36.5)	33 (26.0)	
English-dominant	27 (14.1)	9 (7.1)	
Balanced French/English	95 (49.5)	85 (66.9)	
Family type: Number of children (under 20 years)			0.06
1	42 (21.9)	45 (35.4)	
2	95 (49.5)	52 (40.9)	
≥3	55 (28.6)	30 (23.6)	
Family type: Age group of children ^6^			0.03 *
Preschool age	29 (15.1)	33 (26.0)	
School age	121 (63.0)	63 (49.6)	
Mixed	42 (21.9)	31 (24.4)	
Family type: Gender of children			<0.001 *
Boys only	43 (22.4)	54 (42.5)	
Girls only	55 (28.6)	30 (23.6)	
Mixed	94 (49.0)	43 (33.9)	

^1^ Participants combined from Alberta and Saskatchewan. Classified province of residence from the question presented in the survey. ^2^ NA = Not applicable, as the participant did not respond. ^3^ All responses excluding married and non-separated: unmarried and never legally married, separated but still legally married, divorced, widow or widower. ^4^ Calculated from the last three digits of postal codes declared by the participant. ^5^ Language difference calculated using the average of self-declared spoken, written, and understood competency scores in French and English, followed by subtracting the average English score from the average French score. Positive values represent French-dominant, negative values represent English-dominant, and a score of zero represents balanced English/French levels. ^6^ Preschool age is considered to be <6 years old. School age is considered to be 6 years and above. * *p*-value ≤ 0.05 is statistically significant. *p*-values were calculated using the chi-square test for independence.

**Table 2:** High-priority post-pandemic healthcare and well-being needs for Francophone families in Alberta/Saskatchewan, and Manitoba.

Healthcare and Well-Being Needs	AB and SK ^1^ (*n* = 192) (%)	MB ^2^ (*n* = 127) (%)	*p*-Value *	Effect Size ^3^ (Cohen’s *d*)
Access to recreational, athletic, and artistic activities for children and youth ◊	28.6	23.6	0.61	0.06
Access to healthcare professionals in French ◊	21.3	29.9	0.37	0.10
Social activities in French for families ◊	18.2	22.0	0.58	0.06
Access to mental health services in French for adults #	16.7	-	0.25	0.13
Access to mental health services in French for children and youth #	16.1	-	0.67	0.05
Feel part of a Francophone community #	-	17.3	0.30	0.12
Quality education services in French #	-	17.3	0.52	0.07

^1^ Alberta and Saskatchewan. ^2^ Manitoba. ◊ Common healthcare and well-being needs. # Unique healthcare and well-being needs per population group. * *p*-value ≤ 0.05 is statistically significant. Student’s *t*-test conducted to compare group means. No significant values found. ^3^ All results converted to their absolute value.—Indicative of the post-pandemic need not considered a priority for the province group.

**Table 3:** Risk factors associated with high-priority post-pandemic healthcare and well-being needs are shared between Alberta/Saskatchewan and Manitoba.

Sociodemographic Variable ^1^	Access to Recreational Activities for Children and Youth		Access to Healthcare Professionals in French		Social Activities in French for Families	
	OR (95% CI) ^2^	*p*-Value	OR (95% CI)	*p*-Value	OR (95% CI)	*p*-Value
Participant age						
20–35 (reference group) ^3^	-	-	-	-	-	-
36–45	0.82 (0.29–2.31)	0.70	0.59 (0.21–1.70)	0.34	2.26 (0.77–6.68)	0.14
≥46	0.96 (0.48–1.91)	0.91	0.94 (0.46–1.91)	0.86	1.71 (0.75–3.90)	0.20
Education level						
Secondary or less	-	-	-	-	-	-
Undergraduate Degree/ Diploma	1.56 (0.78–3.11)	0.21	2.55 (1.21–5.42)	0.01 *	1.11 (0.51–2.39)	0.80
Graduate	1.11 (0.59–2.10)	0.75	2.81 (1.46–5.39)	0.002 *	0.97 (0.48–1.98)	0.93
Marital status						
Not married	-	-	-	-	-	-
Married	1.08 (0.49–2.36)	0.86	0.91 (0.41–2.01)	0.81	0.77 (0.30–1.98)	0.58
Not born in Canada	0.93(0.51–1.69)	0.81	1.32 (0.70–2.48)	0.39	1.63 (0.85–3.14)	0.15
Urban setting	0.71 (0.32–1.57)	0.40	1.51 (0.65–3.47)	0.34	1.33 (0.59–2.98)	0.49
Support system in the province						
No	-	-	-	-	-	-
Yes/Sometimes	0.89 (0.47–1.68)	0.71	1.04 (0.54–2.00)	0.91	0.65 (0.31–1.37)	0.26
Language level						
English/French balanced	-	-	-	-	-	-
English-dominant	0.72 (0.29–1.78)	0.48	3.17 (1.01–9.93)	0.048 *	1.13 (0.43–3.02)	0.80
French-dominant	0.82 (0.43–1.58)	0.56	1.10 (0.57–2.13)	0.78	1.15 (0.56–2.34)	0.70
Number of children						
1	-	-	-	-	-	-
2	0.72 (0.31–1.64)	0.43	1.40 (0.59–3.31)	0.45	0.43 (0.17–1.07)	0.07
≥3	0.95 (0.34–2.65)	0.92	1.60 (0.55–4.69)	0.39	0.42 (0.14–1.28)	0.13
Age group of children						
School age	-	-	-	-	-	-
Preschool age	1.30 (0.51–3.30)	0.58	1.66 (0.61–4.51)	0.32	0.88 (0.31–2.44)	0.80
Mixed	0.87 (0.40–1.87)	0.71	0.81 (0.37–1.74)	0.58	1.57 (0.68–3.63)	0.29
Gender of children						
Boys only	-	-	-	-	-	-
Girls only	3.00 (1.38–6.54)	0.006 *	0.78 (0.36–1.70)	0.53	0.49 (0.22–1.11)	0.09
Mixed	2.37 (1.02–5.50)	0.04 *	0.59 (0.26–1.31)	0.19	0.70 (0.30–1.62)	0.41

^1^ *n* = 319. Participants were combined from Alberta, Saskatchewan, and Manitoba. ^2^ OR (Odds Ratio) and CI (Confidence Interval). ^3^ The first sociodemographic item in each list is the reference group for logistic regression analysis. * *p*-value ≤ 0.05. Statistically significant.

**Table 4:** Odds Ratios and corresponding 95% Confidence Intervals for logistic regression analysis of specific healthcare and well-being needs in Alberta and Saskatchewan.

Sociodemographic Variable ^1^	Access to Mental Health Services in French for Adults		Access to Mental Health Services in French for Children and Youth	
	OR (95% CI) ^2^	*p*-Value	OR (95% CI)	*p*-Value
Participant age				
20–35 (reference group) ^3^	-	-	-	-
36–45	1.30 (0.27–7.17)	0.76	0.24 (0.06–1.05)	0.06
≥46	0.59 (0.21–1.65)	0.31	0.19 (0.03–1.10)	0.06
Education level				
Secondary or less	-	-	-	-
Undergraduate Degree/ Diploma	0.46 (0.17–1.26)	0.13	0.98 (0.34–2.84)	0.97
Graduate	0.50 (0.18–1.37)	0.18	0.79 (0.26–2.37)	0.68
Marital status				
Not married	-	-	-	-
Married	1.11 (0.37–3.31)	0.85	1.76 (0.59–5.22)	0.31
Not born in Canada	0.68 (0.27–1.71)	0.42	1.86 (0.73–4.78)	0.20
Urban setting	1.48 (0.49–4.46)	0.49	1.70 (0.55–5.27)	0.36
Support system in the province				
No	-	-	-	-
Yes/Sometimes	0.88 (0.34–2.33)	0.80	1.57 (0.59–4.16)	0.37
Language level				
English/French balanced	-	-	-	-
English-dominant	1.43 (0.44–4.69)	0.56	1.71 (0.45–6.47)	0.43
French-dominant	0.64 (0.23–1.75)	0.38	0.78 (0.30–2.09)	0.63
Number of children				
1	-	-	-	-
2	1.28 (0.35–4.74)	0.71	2.95 (0.49–8.93)	0.32
≥3	0.76 (0.13–4.36)	0.76	2.32 (0.38–14.01)	0.36
Age group of children				
School age	-	-	-	-
Preschool age	0.78 (0.17–3.53)	0.74	0.65 (0.11–3.72)	0.63
Mixed	0.56 (0.14–2.29)	0.42	1.12 (0.33–3.87)	0.86
Gender of children				
Boys only	-	-	-	-
Girls only	1.44 (0.39–5.41)	0.93	0.88 (0.22–3.43)	0.89
Mixed	1.06 (0.29–3.90)	0.59	0.91 (0.23–3.57)	0.85

^1^ *n* = 192. Participants were combined from Alberta and Saskatchewan. ^2^ OR (Odds Ratio) and CI (Confidence Interval). ^3^ The first sociodemographic item in each list is the reference group for logistic regression analysis.

**Table 5:** Odds Ratios and corresponding 95% Confidence Intervals for logistic regression analysis of specific healthcare and well-being needs in Manitoba.

Sociodemographic Variable ^1^	Feel Part of a Francophone Community		Quality Education Services in French	
	OR (95% CI) ^2^	*p*-Value	OR (95% CI)	*p*-Value
Participant age				
20–35 (reference group) ^3^	-	-	-	-
36–45	0.35 (0.07–1.84)	0.21	5.22 (0.86–31.66)	0.07
≥46	1.28 (0.24–6.89)	0.77	3.78 (0.45–31.42)	0.22
Education level				
Secondary or less	-	-	-	-
Undergraduate Degree/ Diploma	0.71 (0.12–4.35)	0.71	3.05 (0.26–35.67)	0.37
Graduate	0.90 (0.18–4.43)	0.89	3.38 (0.34–33.76)	0.30
Marital status				
Not married	-	-	-	-
Married	0.40 (0.08–1.94)	0.26	NA	NA
Not born in Canada	0.98 (0.27–3.56)	0.98	1.99 (0.57–6.95)	0.28
Urban setting	0.84 (0.23–3.10)	0.79	1.26 (0.25–6.46)	0.78
Support system in the province				
No	-	-	-	-
Yes/Sometimes	1.43 (0.24–8.37)	0.70	2.02 (0.37–11.07)	0.42
Language level				
English/French balanced	-	-	-	-
English-dominant	0.67 (0.11–4.09)	0.67	0.99 (0.14–7.14)	0.99
French-dominant	0.47 (0.05–4.18)	0.50	0.54 (0.05–5.43)	0.60
Number of children				
1	-	-	-	-
2	3.45 (0.80–14.86)	0.10	3.15 (0.51–19.46)	0.22
≥3	2.42 (0.28–21.07)	0.43	7.97 (0.95–66.89)	0.06
Age group of children				
School age	-	-	-	-
Preschool age	1.84 (0.38–8.97)	0.45	7.76 (1.16–51.85)	0.04 *
Mixed	1.95 (0.32–11.79)	0.47	2.60 (0.63–10.65)	0.19
Gender of children				
Boys only	-	-	-	-
Girls only	1.16 (0.34–3.96)	0.81	1.92 (0.45–8.23)	0.38
Mixed	0.17 (0.03–0.88)	0.03 *	0.81 (0.20–3.19)	0.76

^1^ *n* = 127. ^2^ OR (Odds Ratio) and CI (Confidence Interval). ^3^ The first sociodemographic item in each list is the reference group for logistic regression analysis. *: *p*-value ≤ 0.05. Statistically significant. NA: OR and CI removed due to perfect separation.

**Figure A1:**
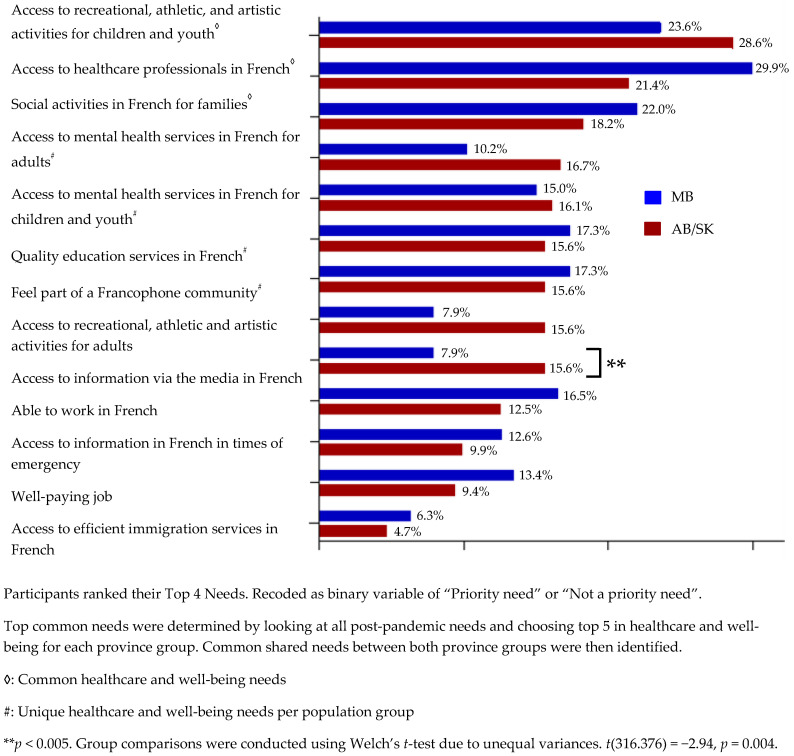
Frequency of post-pandemic healthcare and well-being needs in Alberta/Saskatchewan, and Manitoba grouped populations.

## Data Availability

The original contributions presented in this study are included in the article. Further inquiries can be directed to the corresponding author.

## Abbreviations

The following abbreviations are used in this manuscript:

FF	Francophone families
PPHW	Post-pandemic healthcare and well-being
AB	Alberta
SK	Saskatchewan
MB	Manitoba
